# Distribution of Bathyarchaeota Communities Across Different Terrestrial Settings and Their Potential Ecological Functions

**DOI:** 10.1038/srep45028

**Published:** 2017-03-21

**Authors:** Xing Xiang, Ruicheng Wang, Hongmei Wang, Linfeng Gong, Baiying Man, Ying Xu

**Affiliations:** 1 State Key Laboratory of Biogeology and Environmental Geology, China University of Geosciences, Wuhan, 430074, China; 2 Laboratory of Basin Hydrology and Wetland Eco-restoration, China University of Geosciences, Wuhan, 430074, China; 3 State Key Laboratory Breeding Base of Marine Genetic Resources, Key Laboratory of Marine Genetic Resources, Third Institute of Oceanography, SOA, Key Laboratory of Marine Genetic Resources of Fujian Province, Xiamen, 361005, China.

## Abstract

High abundance and widespread distribution of the archaeal phylum Bathyarchaeota in marine environment have been recognized recently, but knowledge about Bathyarchaeota in terrestrial settings and their correlation with environmental parameters is fairly limited. Here we reported the abundance of Bathyarchaeota members across different ecosystems and their correlation with environmental factors by constructing 16S rRNA clone libraries of peat from the Dajiuhu Peatland, coupling with bioinformatics analysis of 16S rRNA data available to date in NCBI database. In total, 1456 Bathyarchaeota sequences from 28 sites were subjected to UniFrac analysis based on phylogenetic distance and multivariate regression tree analysis of taxonomy. Both phylogenetic and taxon-based approaches showed that salinity, total organic carbon and temperature significantly influenced the distribution of Bathyarchaeota across different terrestrial habitats. By applying the ecological concept of ‘indicator species’, we identify 9 indicator groups among the 6 habitats with the most in the estuary sediments. Network analysis showed that members of Bathyarchaeota formed the “backbone” of archaeal community and often co-occurred with Methanomicrobia. These results suggest that Bathyarchaeota may play an important ecological role within archaeal communities via a potential symbiotic association with Methanomicrobia. Our results shed light on understanding of the biogeography, potential functions of Bathyarchaeota and environment conditions that influence Bathyarchaea distribution in terrestrial settings.

Many uncultured archaeal groups have been unveiled by culture-independent methods, such as the Marine Benthic Group D (MBGD), Miscellaneous Crenarchaeota Group (MCG, recently assigned into a novel archaeal phylum Bathyarchaeota) and Terrestrial Miscellaneous Euryarchaeota Group (TMEG). Since the first observation of MCG via DNA sequencing in 2003^1^, MCG have been detected in different marine^2^,^3^,^4^ and terrestrial^5^,^6^,^7^ environments and dominated in various sediments^8^,^9^ and soils^10^,^11^ as indicated by 16S rRNA clone libraries.

Although MCG has been detected in various ecosystems, it is still difficult to elucidate their physiological functions and ecological roles due to their slow growth rate and lack of pure cultures. The detection of archaeal BacteriochlorophyII synthase gene suggested that the member of MCG may be able to fix CO_2_ via photosynthesis^12^. However, evidence about their heterotrophic life is emerging recently. For instance, MCG has been shown to be able to assimilate ^13^C-labeled acetate^13^ and sedimentary organic carbon^14^ as indicated by DNA-SIP and bulk isotope of intact membrane lipids, respectively. Another line of evidence of its heterotrophic lifestyle is that detection of the gene encoding extracellular protein-degrading enzyme by single-cell sequencing^15^. Besides, some assembled genomes of MCG showed that they may capable of hydrolyzing extracellular plant-derived carbohydrates^16^, utilizing various methylated compounds for methylotrophic methanogenesis^17^ and fermenting organic substrates for acetogenesis^18^. Furthermore, the expression of the gene involved in degradation of aromatic compounds in MCG has been demonstrated with protocatechuate as the substrate^19^.

Phylogenetically, MCG is rather divergent. Totally 21 subgroups were identified and the similarity between the most distant sequences is 75%^20^. Indicator value analysis indicates that some MCG subgroups appear to adapt well to specific environments, such as subgroups 1, 3 and 8 to saline sediments, subgroups 5a, 5b, 7, 9 and 11 to freshwater sediments^20^. Moreover, salinity is demonstrated to be the main factor controlling the adaptive evolution of subgroups as indicated by ancestor state reconstruction analysis^20^.

Despite these advances, the geographic distribution pattern of MCG subgroups among terrestrial habitats remains largely unknown. One reason is that, systematic and comprehensive evaluation about abundance and diversity of MCG subgroups in terrestrial environments is still sparse. On the other hand, multiple factors may affect the structures of MCG communities which make it difficult to clearly picture the general distribution pattern of MCG on large scale. For example, abundant and diverse MCG lineage is detected in acidic and neutral coastal sediments^21^, contrarily only a small amount of MCG is observed in alkaline lagoon sediments^7^. Temperature also impacted the abundance of MCG as indicated by mesocosm experiments with high arctic peat soils which showed an increase of temperature will decrease the relative abundance of MCG members^22^.

With the accumulation of DNA sequencing data from various terrestrial environments, it is highly possible to further understand the distribution pattern of MCG on large scale via bioinformatics analysis. Thus to fully address the distribution pattern of MCG on large ecological scale, archaeal clone libraries were firstly constructed with peat sediments from the acidic Dajiuhu Peatland, central China. Subsequently phylogenetic analysis was conducted by pooling sequences from other terrestrial locations that were available on July 2015. Our results provide new insights into overview on the distribution pattern of MCG across terrestrial ecosystems, their correlation with environmental conditions and their ecological functions.

## Results

### Widespread distribution of MCG lineages

**Figure 1:**
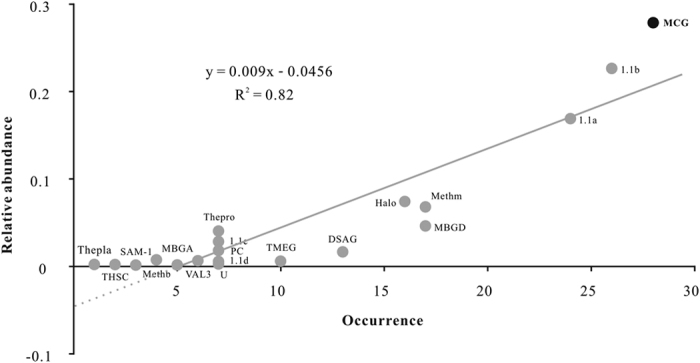
Species abundance distribution (SAD) pattern of archaeal lineages in the libraries analyzed. Abbreviations: 1.1a: Thaumarchaeota 1.1a; 1.1b: Thaumarchaeota 1.1b; 1.1c: Thaumarchaeota 1.1c; 1.1d: Thaumarchaeota 1.1d; DSAG: Deep Sea Archaeal Group; Halo: Halobacteria; MBGA: Marine Benthic Group A; MBGD: Marine Benthic Group D; MCG: Miscellaneous Crenarchaeota Group; Methb: Methanobacteria; Methm: Methanomicrobia; PC: Peat Crenarchaeota; SAM-1: South African Gold Mine Euryarchaeota Group; Thpla: Thermoplasmata; Thepro: Thermoprotei; THSC: Terrestrial Hot Spring Crenarchaeota group; TMEG: Terrestrial Miscellaneous Euryarchaeota Group; VAL3: Val Kotinen lake clade III group; U: unclassified archaeal group.

A total of 890 archaeal OTUs (with 97% cutoff) from 51 sites (Supplementary Table S1) around the world were classified into 19 lineages (Supplementary Fig. S1), whose mean relative abundance was plotted against their occurrence (number of sites in which given lineage was found) (Fig. 1). A significant positive correlation (R^2^ = 0.82) between mean relative abundance and occurrence (Fig. 1) suggested that broadly-occurred archaeal lineages are more abundant than those with restricted occurrence. MCG was the most frequently observed and abundant archaeal group in terrestrial environments. It was detected in 28 sites and accounted for 27.8% of the total archaeal sequences (Fig. 1) with a relative abundance of 0.7–82.1% among those sites.

### Diversity of MCG lineage

The numbers of clone libraries and MCG 16S rRNA sequences varied from one site to another (Table 1). Specifically the number of clone libraries in lagoon was the highest, whilst the number of MCG 16S rRNA sequences was highest in peat sediments. Both the numbers of clone libraries and sequences were the lowest in samples from hot springs (Table 1). However, little difference was observed in Shannon-Wiener indices of MCG among peat sediments, mangrove soils and estuary sediments. The lowest PD index was observed within soils and the highest was in mangrove soils (Table 1).

Different from the biodiversity indexes of Shannon-Wiener and PD, PSV quantifies the phylogenetic relatedness of all species in a community. Observed PSV (Table 1) was remarkably different among habitats. The highest value of PSV in soils indicated a distant phylogenetic relatedness which means a phylogenetic overdispersion. Low PSV values in hot springs, estuary sediments and mangroves suggested a close phylogenetic relatedness which means a phylogenetic clustering. Overall, the mean observed PSV value (0.56) was significantly lower than those given by two null hypothesis models Null 1 (0.64, p < 0.05) and Null 2 (0.61, p < 0.05). These results indicated that coexisting taxa within habitats were more closely related (phylogenetic clustering) than expected by chance.

**Figure 2:**
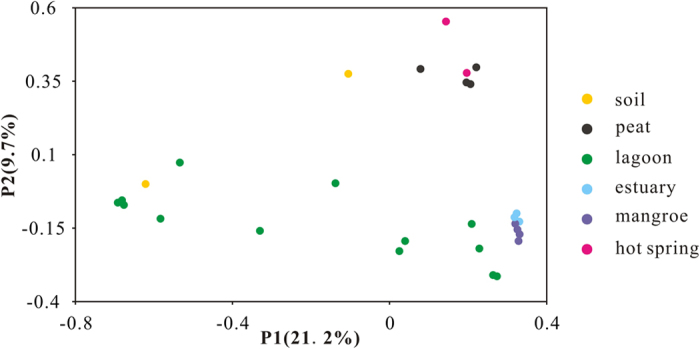
Princlipal coordinate anaylsis (PCoA) of 28 MCG clone libraries based on UniFrac-weighted distance matrix.

Beta diversity of these clone libraries were analyzed with weighted PCoA according to phylogenetic community similarity. Principle coordination 1 (P1) and 2 (P2) explained 21.2% and 9.7% of the total variance, respectively (Fig. 2). MCG communities showed a high specificity to habitats, samples from a specific habitat clustered together well except those from lagoons with a scattering distribution. The high specificity was further strongly supported by permutation manova test (R^2^ = 0.53, P < 0.01). It is interesting to see that phylogenetic community composition of estuary sediments and mangrove soils, peat sediments and hot springs were close related to each other, respectively (Fig. 2).

### Phylogenetic analysis and community compositions of MCG lineages

**Figure 3:**
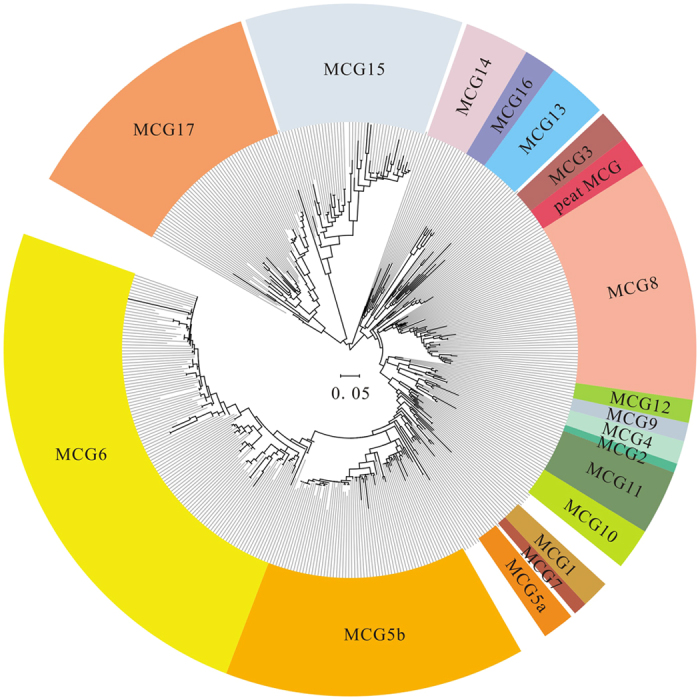
Phylogenetic tree of MCG 16S rRNA genes from different environments was constructed by Maximum Likelihood method under the optimal GTR + G mode. The scale bar represented 5% estimated sequence divergence. The colored leaves presented different MCG subgroups, and uncolored leaves showed unclassified sequences.

Totally 1456 MCG sequences (353 OTUs, at the threshold of 97% similarity) were used to construct MCG phylogenetic tree (Fig. 3). The MCG phylogenetic tree was classified into 18 subgroups with intergroup identity ranging from 82% to 96%. Notably, new cluster peat MCG (pMCG) displayed high stability with a bootstrap value of 100%. However, about 1.2% of MCG sequences (17 sequences) still remained unclassified (uMCG).

**Figure 4:**
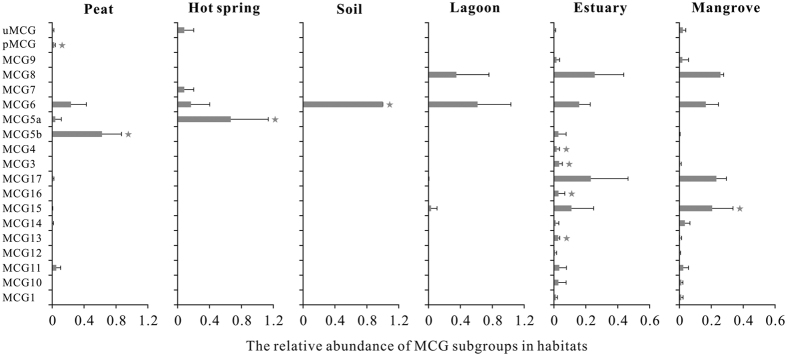
Mean relative abundance of MCG subgroups within different habitats. The error bar represented variance, and asterisks showed indicator groups with a significant threshold of p = 0.05.

As indicated by phylogenetic analysis (Fig. 3), MCG 6 and MCG 5b dominated the MCG lineages with a relative abundance of 38.3% and 20.0%, respectively, followed by MCG 8, MCG 17 and MCG 15. The remaining 13 subgroups contributed <3% of the total sequences (Supplementary Table S2). Overall, MCG communities showed high similarities both in group number and the dominant groups in a specific habitat (Fig. 4). For example, subgroup number ranged from 4 to 6 and the dominant subgroup was MCG 5b in all peat samples (Fig. 4). However, both the number of MCG subgroups and community composition varied significantly in estuaries. Totally 16 subgroups have been detected and MCG 8 and MCG 17 dominated estuarine MCG communities with a relative abundance of 25.6% and 23.1%, respectively. Fourteen MCG subgroups were observed in mangrove soils. Among the MCG subgroups, subgroup 8 and 15 were detected from various environments such as lagoons, estuary sediments and mangroves. In addition, MCG subgroup 6 was the sole subgroup detected in soil samples.

Indicator value (IndVal) function analysis identified 9 indicator groups from all habitats investigated (Fig. 4). Estuary sediments had 4 indicator groups, followed by peat sediments (2), mangrove soils (1), hot springs (1) and soils (1). No indicator group was observed in lagoon samples (Fig. 4). Furthermore, MCG subgroup 3, 5a, 5b, 13 and peat MCG with high IndVal values (0.83–1.0) suggested a high specificity to their habitats. The other indicator MCG subgroups showed a moderate specificity to their habitats with IndVal values of 0.43–0.67. IndVal value of MCG 6 was rather low (0.43) due to its extensive distribution in various environments. In contrast, peat MCG lineage was exclusively detected in peat habitat with a relative abundance of 2.5% with the highest IndVal value of 1.0 (Fig. 4).

### Relationship between MCG community structure and environmental factors

We tested the relationship between MCG phylogenetic communities and environmental factors including pH, salinity, temperature, TOC and TN using permutational analysis of variation. Results indicated that salinity was the strongest environmental force (R^2^ = 0.39, P < 0.01), which explains 39% of variation of MCG assemblages. Temperature (R^2^ = 0.10, P = 0.02) and TOC (R^2^ = 0.08, P = 0.02) also had significant influence on MCG community structure, accounting for 10% and 8% of the variation, respectively. The remaining environmental factors did not show significant impact on MCG groups and contributed to only 6% total variance of UniFrac matrix.

**Figure 5:**
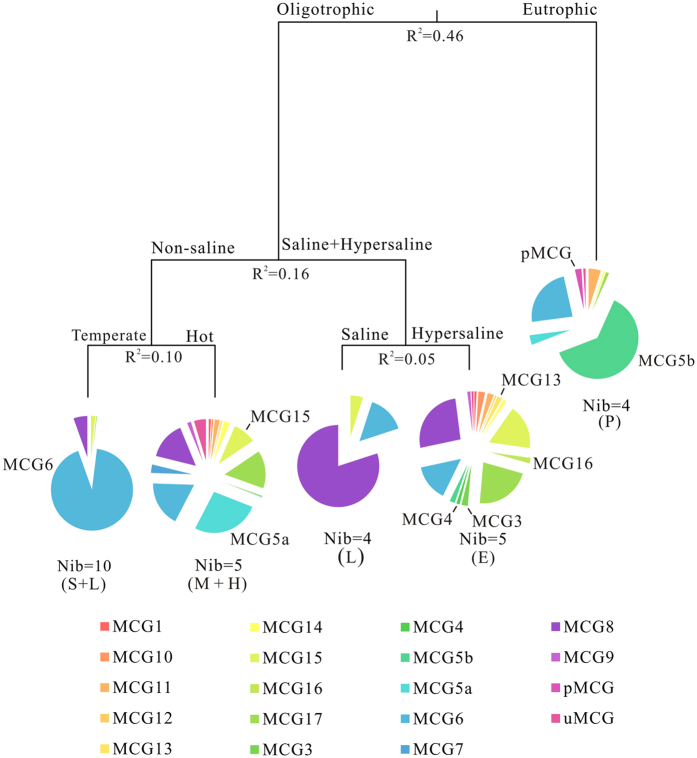
Multivariate regression tree analysis of correlation between MCG subgroups and environmental factors. The pie charts indicated composition and relative abundance of each MCG subgroup in each leaf. Letters in parenthesis displayed habitat types for each leaf: P: peatland; E: estuary; L: lagoon; S: soil; M: mangrove; H: hot spring; Nib: number of libraries or studying sites.

The effects of the environment factors on the community structure of MCG subgroups were determined by multivariate regression tree (MRT) analysis. The variance was shown in a 5-leaf tree mainly based on TOC content, followed by salinity and temperature, which can explain 77% of the variance in total (Fig. 5). Samples collected from peat sediments were grouped together by high TOC contents in the sediments, and estuary sediment samples were clustered together by their similar salinity. However, samples from lagoons were split into two groups as large variation of salinity among these sites (Fig. 5 and Supplementary Table S1). In addition, samples collected from soils and hot springs were clustered with samples from low salinity lagoon and mangrove, respectively, which were affected by temperature (Fig. 5).

The pie charts in MRT showed the relative abundance of MCG subgroups in each leaf. Each leaf was specified by Indicator groups identified by IndVal index, which were mainly responsible for separation of the topology of the MRT. For example, MCG subgroup 5b and pMCG lead to the separation of MCG in peat sediments and in other samples. Similarity, MCG subgroups 3, 4, 13 and 16 resulted in the splitting of MCG in estuary sediments and samples in lagoons with high salinity.

### Interrelation between MCG and other archaeal lineages

**Figure 6:**
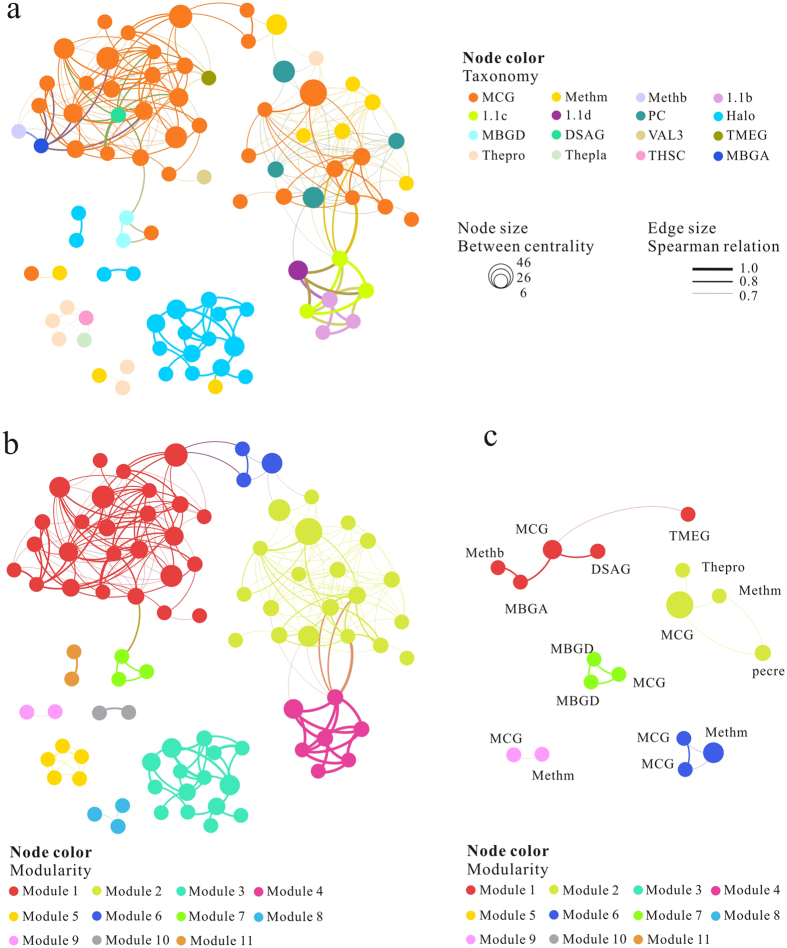
Network analysis of archaeal OTUs in terrestrial settings based on Spearman correlation. Nodes represented archaeal OTUs (90% cutoff) and the node size is proportional to between centrality. Edges between nodes showed significant (P < 0.01) Spearman correlation. (**a**) Nodes were colored by archaeal taxonomy, (**b**) Same network as a, but the node was dyed according to modularity. (**c**) Sub-network of OTU clustering by modularity, only sub-network related with MCG were presented.

A total of 99 archaeal OTUs from 51 sites with a cutoff of 90% were subjected to the co-occurrence network interface analysis to explore potential commensalism that MCG may involve in. The network of terrestrial archaea consisted of 88 nodes and 269 edges among which 40% (35 out of 88) nodes and 68% (184 out of 269) edges were composed by MCG (Fig. 6a). The network topology of terrestrial archaea was characterized by a diameter of 5, average path length of 1.62 and 437 shortest paths. The individual node with top 10 network indices were affiliated with MCG, Halobacteria, 1.1b, peat Crenarchaeota and Methanomicrobia, respectively (Table 2). Furthermore, these network indices of MCG nodes were much higher than those of others archaeal groups, particular for centrality and degree. These results suggested that MCG lineages possess pivotal ecological function among archaeal groups.

The network showed modular structure with a modular index 0.67. The network was classified into 11 modules (Fig. 6b). Modules could be considered as ecological niches (Fisher’s test, P < 0.001) in which OTUs were closely connected to each other and connectivity was significantly higher than those between modules. For example, modules 2, 5 and 8 represented sub-networks of peat sediments, hot springs and saline lakes, respectively. MCG was observed in 5 out of the 11 modules (Fig. 6b). Co-occurrence association was observed between MCG and other archaeal groups such as DSAG, MBGD, peat Crenarchaeota, Thermoprotei, and VAL3 (Fig. 6c). It should be noted that, the co-occurrence of MCG and Methanomicrobia were detected in 3 modules out of 5 containing MCG modules indicating a rather close ecological association (Fig. 6c). Members of Methanomicrobia which co-occurred with MCG were affiliated with *Methanosaeta, Methanoregula, Methanocella, Methanoperedens* and uncultured Methanomicrobiaceae.

## Discussion

MCG is a ubiquitous archaeal group in terrestrial environments. It occurred in 28 of 51 sites investigated and accounted for 27.8% of total archaeal sequences analyzed. MCG widely distributed across various terrestrial habitats including hot springs, soils and lagoons with the relative abundance of MCG ranging from 0.7% to 80.0%. Furthermore MCG dominated the archaeal communities in peat sediments, mangroves and estuary sediments. Compared with previous studies, which found that MCG accounted for 35–80% of archaeal communities in peat sediments^8^, 10–100% in high mountain lake^23^, and 43% in southern red soil^11^, our result provided an overall view of terrestrial distribution of MCG group on a large scale. Wide distribution and relatively high abundance strongly suggest that MCG is an important archaeal group in terrestrial ecological process.

Despite of ubiquitous distribution of MCG in terrestrial environment, some MCG subgroups show well-adapted and high specificity to certain habitats, indicating a close relationship between MCG and environmental conditions. This made them suitable to serve as ecological indicator for specific habitats. In this study, nine indicator subgroups were identified based on indicator value function analysis, and at least one indicator subgroup was observed in most habitats investigated except lagoons.

Previously, salinity has been shown to be a driving force that shapes archaeal communities both within a specific estuary habitat^24^ and on large-scale aquatic habitats^25^. The phylogenetic communities of MCG also showed a close relationship with salinity which significantly architects the composition and abundance of MCG as indicated by MRT. In addition to that, salinity was also found to be an evolutionary barrier that leads to the diversification of MCG between marine and freshwater sediments^20^. This is highly consistent with our observation that salinity was the most significant factor affecting MCG phylogenetic structure.

It is demonstrated that TOC greatly constrains the distribution and diversity of heterotrophs due to their high dependence on TOC for carbon source^26^,^27^. It should be noted that in our research TOC also significantly shaped the distribution of MCG on a large scale. Even through the knowledge about MCG life style is still limited, several lines of evidence support the heterotrophic lifestyle of MCG recently^13^,^15^,^18^. Our observation of the significant impact of TOC on MCG distribution may mount another line of heterotrophic lifestyle of MCG^28^.

Temperature is also an important factor controlling microbial communities. For example, it has been found to be a dominant factor that drives microbial diversity and composition in geothermal springs^29^. Importantly, increase of temperature will decrease the relative abundance of MCG as indicated by both laboratory experiment^22^ and field investigation^7^. Our study also demonstrated the impact of temperature on MCG communities to certain extent.

Compared with restricted distribution of Thermoprotei in hyperthermal environments, the wide distribution of MCG across terrestrial environments indicates that MCG have adapted to various environments and potentially have versatile metabolic pathways for survival. Co-occurrence network analysis indicates the dominance of MCG in nodes, edges and modules (Fig. 6b). Moreover, individual nodes with top 10 highest values of network indices are mainly affiliated with MCG. All of these suggest that MCG is the most abundant and widespread archaeal group and plays an important role in construction of archaeal network. Likely, MCG serve as “keystone species” which functions to maintain the stability and adaptability of archaeal community^30^.

Furthermore, the co-occurrence pattern also reveals some of interesting ecological patterns among archaeal divisions which have not been well revealed previously. For example, MCG co-occurred with the members of 1.1a, 1.1b, DSAG, MBGD, peat Crenarchaeota, Thermoprotei, and VAL3. These co-occurrences suggest a preference of similar environmental conditions of these archaeal groups or a commensalism relationship between them. However, the currently available data do not permit us to distinguish these two possibilities at this point.

Notably, members of MCG co-occur more often with the Methanomicrobia (including *Methanosaeta, Methanoregula, Methanocella, Methanoperedens* and Methanomicrobiaceae) than with other archaeal groups, which indicates a high possibility of a syntrophic association between these two groups. We proposed that acetate may be response for this close association between those two groups. Acetate has been demonstrated to be important organic substrate for growth and methane production of acetoclastic *Methanosaeta*^31^. Meanwhile, acetate is also a necessary growth factor for hydrogenotrophic *Methanoregula*^32^, *Methanocella*^33^ and Methanomicrobiaceae^34^. Importantly, some MCG members were demonstrated to possess a gene encoding acetate kinase^18^, which is involved in acetate production. This may explain the mechanism of symbiosis between MCG and Methanomicrobia. In fact the close physical association between the members of MCG and Methanomicrobia (*Methanosaeta*) has been demonstrated by fluorescence *in situ* hybridization (FISH) in active sludge^35^.

Due to the high specificity to a given environment, indicator MCG subgroups may play important roles in biogeochemical processes under specific conditions. For example, MCG subgroup 15 (the indicator group of mangrove soils) showed a high abundance in coast and marine environments and their genomic data indicated that they are highly capable of extracellular protein degradation, acetate-producing and dissimilatory nitrite reduction^16^. MCG subgroup 6 (indicator group for soils) may also capable of degrading plant-derived carbohydrate under the anaerobic condition besides the functions proposed for MCG subgroup 15^16^. As indicator groups of estuary sediments, MCG subgroup 3 is proposed to be methanogens and contributes to global methane cycling^17^. Subgroup 16 (indicator group for estuary) could utilize a variety of organic substrate via fermentation to produce acetate^18^ which could be subsequently utilized by various microbial groups^36^.

Lack of available physiologic and genomic information renders it difficult to elucidate the ecological function of the remaining 5 indicator MCG subgroups (MCG 4, 13, 5a, 5b and peat MCG). Based on their phylogenetic distance with the groups whose genomic data are available, the functions of some of these subgroups are speculated. For MCG subgroup 13, we hypothesize that they play a significant role in degrading various organic compounds such as protein, starch, and persistent aromatic compounds according to their close phylogenetic relationship with MCG 16 (Fig. 3). Because they are phylogenetically related to MCG subgroup 6 and 7 (Fig. 3), MCG subgroup 5a and 5b might degrade carbohydrate, protein debris and reduce nitrite to ammonia. As they show no close relationship with other MCG subgroups, the ecological functions of MCG subgroup 4 and peat MCG cannot be speculated.

## Materials and Methods

### Site description and sampling

The Dajiuhu Peatland (31°28′50″N, 110°00′90″E) locates in northwest Hubei province and is characterized by typically sub alpine monsoon climate. The annual temperature and precipitation were 7.2 °C and 1,560 mm, respectively. The organic carbon was abundant due to the low temperature condition and high ground water level^37^.

Four peat sediment samples were collected with 50 ml sterilized centrifuge tubes along a profile in Sep, 2012 and transported to the geomicrobiology lab in China University of Geosciences (Wuhan) on dry ice immediately. Samples for molecular analysis were stored at −80 °C. Samples for physicochemical analysis were freezing dried and grinded into powder.

### Physicochemical analysis

Homogeneous suspension was obtained by mixing the grinded peat sediments with ultra-pure water with a ratio of 1:2.5 (w/v) via stirring with a glass rod for 10 min. After sitting quietly for 30 min, the pH of supernatant was measured three times for each sample and the mean value of three measurements was reported. The total organic carbon (TOC) and total nitrogen (TN) in sediment samples were analyzed with Elemental Analyzer (Elementar Vario EL III) with a precision of ±0.4%^38^.

### Clone library construction of archaeal 16S rRNA

Genomic DNA was extracted with Fast DNA spin kit for soil (MP, USA) according to manufacture’s instructions. Extracted DNA was stored at −80 °C after the concentration was measured with Nanodrop ND-2000 spectrophotometer (Thermofisher, Germany). Archaeal 16S rRNA gene fragments were amplified with the primer set A21F and A958R^39^ by the following steps: 94 °C for 5 min, 36 cycles of 94 °C for 30 s, 58 °C for 45 s, 72 °C for 1 min, and final extension at 72 °C for 10 min. PCR mixture (20 μl) contained 2.0 μl 10 × PCR buffer, 2.0 μl Mg^2+^ (25 mM), 2.0 μl dNTP (2.5 mM), 0.5 μl of each primer (20 mM), 0.25 μl of Taq DNA polymerase (5 U/μl), 1 μl of BSA (20 μg/μl) (TaKaRa, Dalian, China), 2 μl of template DNA (20 ng/μl) and 9.75 μl of PCR water. All the PCR reactions were run on a Master Cycler 5331 Gradient PCR (Eppendorf, Hamberg, Germany).

PCR product was visualized with 1% agarose gel. Target bands were excised and recovered with QIAquick gel extraction kit (Qiagen, Germany) according to the manufacturer’s instructions. Purified PCR product fragments were ligated into pMD18-T plasmid vector (TaKaRa, Dalian, China) and transformed into the *Escherihcia coli* JM109 cell (TaKaRa, Dalian, China). Transformants were identified based on blue-white screening on LB media containing 100 μg/ml of ampicillin. The presence of the insert was screened directly with M13F and M13R vector primers and positive clones were sequenced with A21F primer by Genscript Company (Nanjing, China). Possible vector contamination was checked using NCBI Vecscreen (http://www.ncbi.nlm.nih.gov/tools/vecscreen/). Totally 216 valid sequences were acquired from Dajiuhu peat sediments which are deposited in GenBank under accession numbers of KM514090-KM514305. All the sequences were pooled together as one clone library to increase the representativeness.

### Selection criteria for archaeal 16S rRNA sequence through NCBI nr database

To discern the distribution pattern of MCG communities across terrestrial ecosystems and the association between MCG communities and environmental parameters, we collected archaeal 16S rRNA sequences available in Genbank database. Only the sequences of 1) that were amplified with the universal primer set (A21F and A958R); 2) that contained no nucleotide ambiguities and whose lengths were >700 bp; 3) whose sampling site were well defined were used.

### Construction of phylogenetic tree

ClustalW program^40^ was used to align the 5226 archaeal 16S rRNA sequences from 51 investigated sites and Mothur software was used to calculate distance matrix and cluster sequences^41^. Those sequences were clustered into Operation Taxonomy Unit (OTU) at the identity level of 97% (890 OTUs) for diversity analysis and 90% (237 OTUs) for network analysis. The selected 890 OTUs (97% similarity) were used to construct archaeal taxonomy tree based on Maximum likelihood method by GRT + G + T mode with Mega 6.0 software^42^. Among 890 archaeal OTUs, 353 MCG OTUs were selected to construct MCG phylogenetic tree. The best phylogenetic tree was constructed with GTR + G2 mode with the bootstrap 1000 and was drawn by iTOL^43^.

### Statistical analysis

Phylogenetic diversity (PD)^44^ and Shannon-Wiener index^45^ were used to characterize microbial diversity. PD is the sum of phylogenetic branch length within a habitat^44^. Mean PD value of 1000 random subsamples were used to correct unequal sequence numbers between habitats. The Shannon-Wiener index indicates the number and abundance of species within a habitat which positively correlates with richness and evenness, and is sensitive to the abundance variation of rare species^46^.

UniFrac was conducted to analyze beta diversity which quantifies community similarity based on phylogenetic relativeness^47^. Principle coordinate analysis (PCoA) was used to visualize beta diversity obtained from UniFrac distance matrix. Permutation manova based on 1000 permutations with R (version 3.2.2)^48^ package vagan (version 2.4-1)^49^ was used to explain phylogenetic variance from UniFrac distance.

Species abundance distribution (SAD) pattern and ecological importance of MCG lineage were estimated according to the relative abundance of archaeal lineages. A multivariate regression tree (MRT) was carried out by R package mvpart (version 1.6-2)^50^ to determine the relationship between MCG subgroups and environmental factors.

Phylogenetic structure was estimated with phylogenetic species variability (PSV)^51^ which based on species presence/absence and theirs phylogenetic relativeness. This metrics is between 0 and 1, and asymptotically approach 0 as relatedness increases. Comparison between observed PSV and null values from different randomized models were conducted by R package picante (version 1.6-2)^52^ to statistically test whether MCG community composition of different habitats is more closely related or distantly related than expected by chance.

Correlation-based method^53^ as well as probabilistic graphic model method^54^ is a common approach to explore underlying mechanism of microbial ecological process. Despite of the deficiency in the information of control and dependency, correlation-based method shows direct relatedness between (environmental and biological) variables and is widely used to study potential interactions between microorganisms^20^,^55^. In this study we analyzed correlation between OTUs affiliated with MCG and other archaeal lineages using correlation-based method based on Spearman coefficient^56^. We calculated all possible Spearman rank correlation between those OTUs (at the threshold of 90% identity) with more than 5 sequences (99 OTUs). We removed those poor representative OTUs and reduced network complexity, facilitating the determination of core archaeal community. Valid co-occurrence event with rank correlation coefficients above 0.6 and statistical significant below 0.01^57^ were considered to be a robust correlation. The network was characterized by topology indices (diameter, average path length, modularity, number of the shortest path) and indices of an individual node (degree, closeness centrality, between centrality). Specially, modularity was calculated using Louvain algorithm^58^, which include two phases of modularity optimization and community aggravation. Aggravation is repeated until modularity no longer can be increased. All these analysis were performed by R package psych (version 1.6.9)^59^ and Gephi (version 0.8.2 beta) software^60^. Network images were visualized by Gephi software. The correlation between modules and habitats were statistically tested by the Fisher’s test under software R.

## Additional Information

**How to cite this article:** Xiang, X. *et al*. Distribution of Bathyarchaeota Communities Across Different Terrestrial Settings and Their Potential Ecological Functions. *Sci. Rep.* **7**, 45028; doi: 10.1038/srep45028 (2017).

**Publisher's note:** Springer Nature remains neutral with regard to jurisdictional claims in published maps and institutional affiliations.

## Supplementary Material

Supplementary figure and Table

## Figures and Tables

**Table 1:** Diversity index of MCG lineages in different habitats.

Habitat	Nib	N	Shannon-Wiener	PD	PSV
Peat sediment	4	472	3.42 ± 0.61	1.76 ± 0.47	0.56 ± 0.13
Soil^a^	2	130	1.12	0.21	0.86
Hot spring	2	7	2.69	0.30	0.36
Lagoon	13	217	1.87 ± 1.19	0.59 ± 0.47	0.61 ± 0.21
Mangrove soil	4	433	3.68 ± 0.77	2.96 ± 1.08	0.48 ± 0.02
Estuary sediment	3	197	3.56 ± 0.23	2.45 ± 0.40	0.47 ± 0.05

Nib: number of libraries or studying sites.

N: number of clones.

^a^Libraries containing only one OTU were excluded from the calculation of the mean biodiversity index.

**Table 2:** Network indices of the top 10 nodes in the network.

Lineages	Closeness centrality	Lineages	Betweenness centrality	Lineages	Degree
Halo	3.50	MCG	46.0	MCG	20
MCG	3.27	MCG	32.0	MCG	18
MCG	3.18	MCG	30.0	MCG	14
Halo	2.86	MCG	27.0	MCG	14
1.1b	2.73	PC	27.0	MCG	14
Halo	2.60	PC	24.2	MCG	14
MCG	2.50	MCG	22.2	MCG	14
1.1c	2.50	Methm	22.0	Methm	14
Halo	2.50	Halo	20.5	PC	13
MCG	2.43	Halo	18.0	MCG	13
