# Supplementary material for: Distribution of Bathyarchaeota Communities Across Different Terrestrial Settings and Their Potential Ecological Functions
Source: Sci Rep. 2017 Mar 21;7:45028. doi: 10.1038/srep45028 (PMC5359579; doi:10.1038/srep45028)
Supplement: Supplementary figure and Table [file srep45028-s1.doc]

**Distribution of Bathyarchaeota Communities**

# Across Different Terrestrial Settings and Their potential ecological functions

Xing XIANG^1^, Ruicheng WANG^1^, Hongmei WANG^1, 2^, Linfeng GONG^3^, Baiying MAN^1^，Ying XU^1^

^1^State Key Laboratory of Biogeology and Environmental Geology, China University of Geosciences, Wuhan, 430074

^2^Laboratory of Basin Hydrology and Wetland Eco-restoration, China University of Geosciences, Wuhan, 430074

^3^State Key Laboratory Breeding Base of Marine Genetic Resources, Key Laboratory of Marine Genetic Resources, Third Institute of Oceanography, SOA, Key Laboratory of Marine Genetic Resources of Fujian Province, Xiamen, 361005

^&^ The authors contributed equally to this work and should be considered co-first authors

^*^ corresponding author: [hmwang@cug.edu.cn](mailto:hmwang@cug.edu.cn); wanghmei04@163.com

**Table S1 Samples information of 51 archaeal 16S rRNA clone libraries**.

| Sample No. | Physiochemical property | | | | | Size of clone library | Number of MCG sequences | Ecological type | Sampling time | Location | | Reference |
| --- | --- | --- | --- | --- | --- | --- | --- | --- | --- | --- | --- | --- |
|  | pH | Salinity(‰) | T(℃) | TOC(%) | TN(%) |  |  |  |  | latitude | longitude |  |
| 2-5C | 3.8 | 1.0 | 16.1 | 26.64 | 1.85 | 253 | 93 | Peat | February, 2008 | -25.27 | -49.83 | [^1^](#_ENREF_1) |
| 2P | 4.0 | 1.0 | 16.1 | 17.26 | 1.85 | 145 | 113 | Peat | February, 2008 | -26.57 | -51.75 | [^1^](#_ENREF_1) |
| 2S | 3.2 | 1.0 | 16.1 | 25.61 | 1.85 | 144 | 114 | Peat | February, 2008 | -25.89 | -48.96 | [^1^](#_ENREF_1) |
| ALMR | 9.5 | 15.8 | 24.4 | 5.15 | 0.28 | 29 | 0 | Lagoon | May, 2007 | 36.81 | 6.37 | [^2^](#_ENREF_2) |
| ALMSd | 9.5 | 15.8 | 24.4 | 5.15 | 0.28 | 31 | 4 | Lagoon | May, 2007 | 36.81 | 6.37 | [^2^](#_ENREF_2) |
| Arc | 5.5 | 1.0 | 18.1 | 0.10 | 0.02 | 127 | 1 | Soil | - | 35.93 | -84.32 | [^3^](#_ENREF_3) |
| BCJR | 8.8 | 1.1 | 12.4 | 1.18 | 0.09 | 46 | 23 | Lagoon | January, 2007 | 42.03 | 3.18 | [^2^](#_ENREF_2) |
| BCJSd | 8.8 | 1.1 | 12.4 | 1.18 | 0.09 | 93 | 74 | Lagoon | January, 2007 | 42.03 | 3.18 | [^2^](#_ENREF_2) |
| BCMR | 8.8 | 1.5 | 19.0 | 1.17 | 0.07 | 30 | 11 | Lagoon | May, 2007 | 42.03 | 3.18 | [^2^](#_ENREF_2) |
| BCMSd | 8.8 | 1.5 | 19.0 | 1.17 | 0.07 | 32 | 5 | Lagoon | May, 2007 | 42.03 | 3.18 | [^2^](#_ENREF_2) |
| BS | 6.8 | 18.7 | 24.0 | 2.20 | 0.05 | 101 | 49 | Mangrove | - | 37.59 | 126.45 | [^4^](#_ENREF_4) |
| CEH | 6.0 | 283.0 | 19.2 | 0.01 | 0.01 | 108 | 0 | Saline lake | - | 37.09 | 94.04 | [^5^](#_ENREF_5) |
| CKS | 7.4 | 37.1 | 16.5 | 0.85 | 0.13 | 102 | 0 | Saline lake | August, 2003 | 36.42 | 99.07 | [^6^](#_ENREF_6) |
| CKW | 7.4 | 325.0 | 16.8 | 0.01 | 0.01 | 24 | 0 | Saline lake | August, 2003 | 36.42 | 99.07 | [^6^](#_ENREF_6) |
| DN | 4.8 | 1.0 | 18.9 | 0.73 | 0.30 | 25 | 0 | Soil | June, 2010 | 25.85 | 114.95 | [^7^](#_ENREF_7) |
| FRJR | 9.0 | 16.0 | 13.5 | 3.61 | 0.04 | 58 | 8 | Lagoon | January, 2007 | 42.03 | 3.18 | [^2^](#_ENREF_2) |
| FRJSd | 9.0 | 16.0 | 13.5 | 3.61 | 0.04 | 17 | 4 | Lagoon | January, 2007 | 42.03 | 3.18 | [^2^](#_ENREF_2) |
| FRMR | 9.1 | 14.8 | 29.9 | 7.80 | 0.54 | 32 | 3 | Lagoon | May, 2007 | 42.03 | 3.18 | [^2^](#_ENREF_2) |
| FRMSd | 9.1 | 14.8 | 29.9 | 7.80 | 0.54 | 10 | 2 | Lagoon | May, 2007 | 42.03 | 3.18 | [^2^](#_ENREF_2) |
| GH | 9.2 | 32.0 | 15.9 | 0.01 | 0.01 | 94 | 0 | Saline lake | - | 37.01 | 100.58 | [^5^](#_ENREF_5) |
| GL52 | 7.1 | 1.0 | 52.0 | 0.05 | 0.01 | 46 | 0 | Hot spring | August,2009 | 30.87 | 91.61 | [^8^](#_ENREF_8) |
| GL63 | 6.6 | 1.0 | 63.8 | 0.10 | 0.01 | 47 | 0 | Hot spring | August,2009 | 30.87 | 91.61 | [^8^](#_ENREF_8) |
| Sample No. | Physiochemical property | | | | | Size of clone library | Number of MCG sequences | Ecological type | Sampling time | Location | | Reference |
|  | pH | Salinity(‰) | T(℃) | TOC(%) | TN(%) |  |  |  |  | latitude | longitude |  |
| GL64 | 7.2 | 1.0 | 64.0 | 0.10 | 0.01 | 50 | 0 | Hot spring | August,2009 | 30.87 | 91.61 | [^8^](#_ENREF_8) |
| GL81 | 8.2 | 1.0 | 81.2 | 0.10 | 0.01 | 47 | 0 | Hot spring | August,2009 | 30.87 | 91.61 | [^8^](#_ENREF_8) |
| GY | 5.2 | 1.0 | 8.0 | 11.91 | 0.55 | 380 | 0 | Soil | May, 2007 | 29.99 | 93.10 | [^9^](#_ENREF_9) |
| HO28 | 2.5 | 1.0 | 28.0 | 0.26 | 0.02 | 94 | 0 | Hot spring | September, 2009 | 35.24 | 139.02 | [^10^](#_ENREF_10) |
| HO78 | 3.5 | 1.0 | 28.0 | 0.01 | 0.01 | 83 | 0 | Hot spring | September, 2009 | 35.24 | 139.02 | [^10^](#_ENREF_10) |
| JW41 | 6.8 | 1.0 | 41.7 | 0.10 | 0.01 | 48 | 0 | Hot spring | August,2009 | 33.14 | 86.83 | [^8^](#_ENREF_8) |
| JW56 | 6.5 | 1.0 | 56.0 | 0.10 | 0.01 | 55 | 0 | Hot spring | August,2009 | 33.14 | 86.83 | [^8^](#_ENREF_8) |
| LCJR | 7.2 | 4.6 | 7.5 | 2.75 | 0.13 | 102 | 65 | Lagoon | January, 2007 | 36.81 | 6.37 | [^2^](#_ENREF_2) |
| LCJSd | 7.2 | 4.6 | 7.5 | 2.75 | 0.13 | 38 | 4 | Lagoon | January, 2007 | 36.81 | 6.37 | [^2^](#_ENREF_2) |
| LCMR | 6.9 | 3.0 | 21.7 | 3.01 | 0.10 | 39 | 8 | Lagoon | May, 2007 | 36.81 | 6.37 | [^2^](#_ENREF_2) |
| LCMSd | 6.9 | 3.0 | 21.7 | 3.01 | 0.10 | 34 | 6 | Lagoon | May, 2007 | 36.81 | 6.37 | [^2^](#_ENREF_2) |
| MES | 7.7 | 32.0 | 24.0 | 2.30 | 0.10 | 179 | 60 | Estuary | January, 2005 | 15.48 | 73.80 | [^11^](#_ENREF_11) |
| MKC | 4.5 | 10.9 | 24.9 | 4.22 | 0.15 | 397 | 249 | Mangrove | October, 2004 | 19.96 | 110.58 | [^12^](#_ENREF_12) |
| MNT | 7.4 | 8.2 | 24.9 | 1.81 | 0.05 | 112 | 92 | Mangrove | October, 2004 | 19.96 | 110.59 | [^12^](#_ENREF_12) |
| MP | 6.3 | 1.0 | 3.5 | 1.92 | 1.20 | 379 | 0 | Soil | May, 2007 | 29.83 | 92.34 | [^9^](#_ENREF_9) |
| MSA | 5.2 | 7.8 | 24.9 | 2.11 | 0.07 | 114 | 43 | Mangrove | October, 2004 | 19.96 | 110.59 | [^12^](#_ENREF_12) |
| Meadow | 6.6 | 1.0 | 3.4 | 3.17 | 0.25 | 279 | 129 | Soil | June, 2013 | 38.09 | 99.17 | [^13^](#_ENREF_13) |
| PES | 7.4 | 26.0 | 21.5 | 1.52 | 0.10 | 137 | 83 | Estuary | April, 2005 | 22.46 | 111.64 | [^14^](#_ENREF_14) |
| RM26 | 6.8 | 1.0 | 26.2 | 0.10 | 0.01 | 49 | 1 | Hot spring | August,2009 | 32.96 | 86.60 | [^8^](#_ENREF_8) |
| RMS | 7.0 | 1.0 | 3.5 | 2.00 | 0.50 | 55 | 0 | Soil | August,2009 | 32.96 | 86.60 | [^8^](#_ENREF_8) |
| RM55 | 7.1 | 1.0 | 55.6 | 0.10 | 0.01 | 56 | 0 | Hot spring | August,2009 | 32.96 | 86.60 | [^8^](#_ENREF_8) |
| RM64 | 6.9 | 1.0 | 64.1 | 0.10 | 0.01 | 44 | 6 | Hot spring | August,2009 | 32.96 | 86.60 | [^8^](#_ENREF_8) |
| RM45 | 7.2 | 1.0 | 45.1 | 0.10 | 0.01 | 48 | 0 | Hot spring | August,2009 | 32.96 | 86.60 | [^8^](#_ENREF_8) |
| DJH | 4.7 | 1.0 | 15.6 | 47.02 | 2.12 | 216 | 152 | Peat | September,2012 | 31.48 | 110.00 | This paper |
| Sample No. | Physiochemical property | | | | | Size of clone library | Number of MCG sequences | Ecological type | Sampling time | Location | | Reference |
|  | pH | Salinity(‰) | T(℃) | TOC(%) | TN(%) |  |  |  |  | latitude | longitude |  |
| VSS | 7.0 | 1.0 | 19.0 | 3.10 | 0.22 | 25 | 0 | soil | June, 2008 | 29.57 | 106.42 | [^15^](#_ENREF_15) |
| VW | 8.5 | 1.0 | 5.0 | 0.01 | 0.01 | 192 | 0 | freshwarter | From January, 2008 to April, 2009 | 47.05 | 8.59 | [^16^](#_ENREF_16) |
| XCD | 9.2 | 160.0 | 21.0 | 0.01 | 0.01 | 95 | 0 | Saline lake | - | 37.28 | 95.28 | [^5^](#_ENREF_5) |
| ZES | 7.7 | 34.0 | 25.0 | 1.00 | 0.10 | 256 | 54 | Estuary | January, 2005 | 15.41 | 73.85 | [^11^](#_ENREF_11) |
| lp | 8.1 | 1.0 | 8.2 | 1.24 | 0.07 | 29 | 0 | Soil | June, 2010 | 39.16 | 112.78 | [^7^](#_ENREF_7) |

- sampling time remained unknown.

**Table S2. Relative abundance of MCG subgroups**

**in the 28 sites where MCG were detected**

| **Taxonomy** | **Relative abundance(%)** |
| --- | --- |
| MCG1 | 0.34 |
| MCG10 | 0.76 |
| MCG11 | 2.61 |
| MCG12 | 0.27 |
| MCG13 | 0.69 |
| MCG14 | 1.51 |
| MCG15 | 6.66 |
| MCG16 | 0.34 |
| MCG17 | 9.75 |
| MCG3 | 0.69 |
| MCG4 | 0.21 |
| MCG5b | 19.99 |
| MCG5a | 1.79 |
| MCG6 | 38.26 |
| MCG7 | 0.07 |
| MCG8 | 12.57 |
| MCG9 | 1.51 |
| pMCG | 0.82 |
| uMCG | 1.17 |


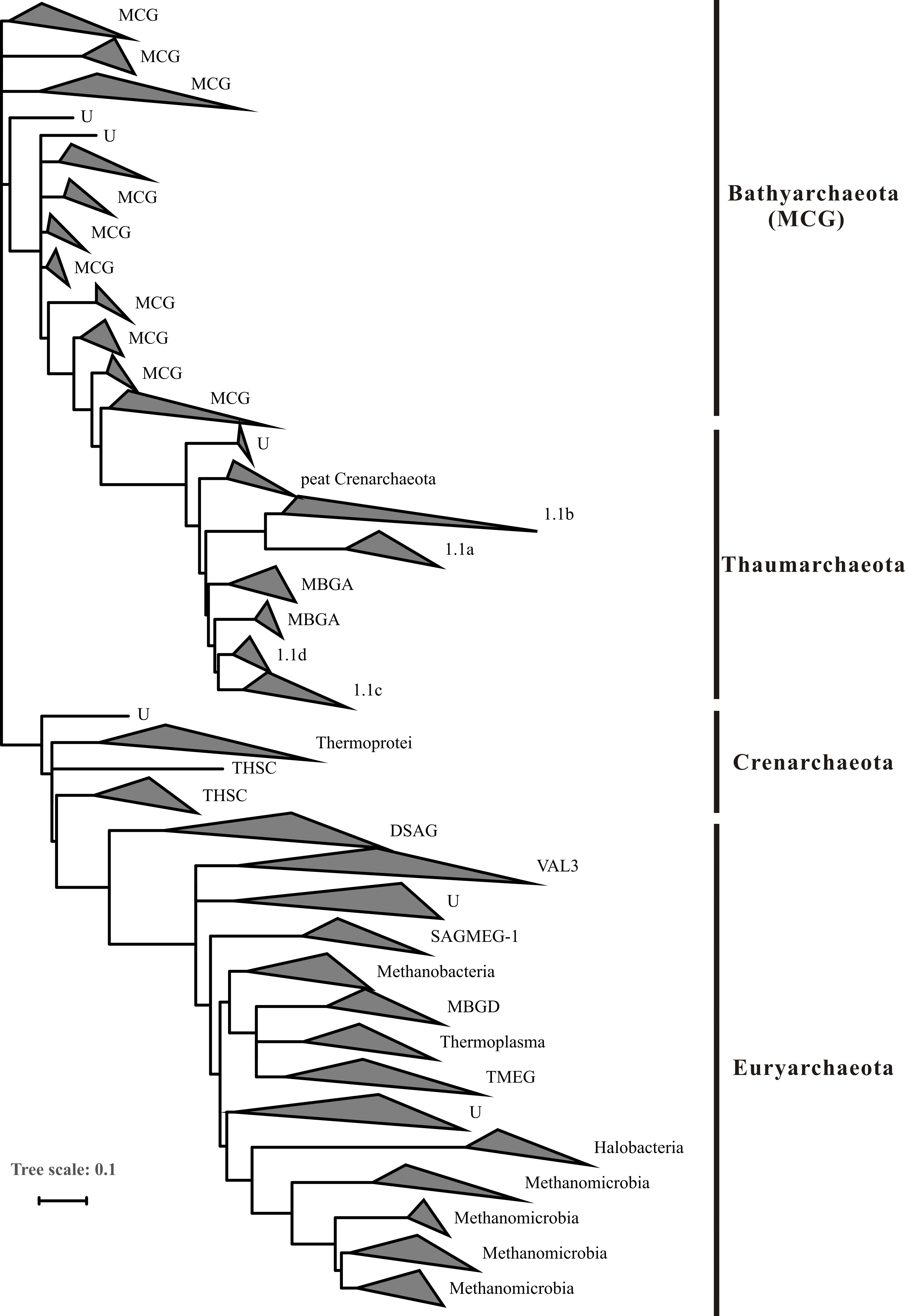


**Fig S1. Phylogenetic tree of archaeal 16S rRNA sequences using MEGA 6.0 by Maximum**

**Likelihood method under GTR + G + I model and was drawn by iTOL**.

Totally 19 archaeal lineages were classified: MCG (Miscellaneous Crenarchaeota Group), peat Crenarchaeota, U (unclassified archaeal lineages), 1.1a (Thaumarchaeota 1.1a ), 1.1b (Thaumarchaeota 1.1b), 1.1c (Thaumarchaeota 1.1c), 1.1d (Thaumarchaeota 1.1d), MBGA(Marine Benthic Group A); Thermoprotei, THSC (Terrestrial Hot Spring Crenarchaeota), DSAG (Deep Sea Archaea Group); VAL3 (Val Kotinen lake clade III group), SAGMEG-1 (South African Gold Mine Euryarchaeota Group), Methanomicrobia; Methanobacteria, Halobacteria; Thermoplasmata, TMEG (Terrestrial Miscellaneous Euryarchaeota Group).

Reference:

1 Etto, R. M. *et al.* Prokaryotic communities of acidic peatlands from the southern Brazilian Atlantic Forest. *Braz. J. Microbiol.* **43**, 661-674 (2012).

2 Llirós, M., Trias, R., Borrego, C. & Bañeras, L. Specific archaeal communities are selected on the root surfaces of Ruppia spp. and Phragmites australis. *Wetlands* **34**, 403-411 (2014).

3 Hansel, C. M., Fendorf, S., Jardine, P. M. & Francis, C. A. Changes in bacterial and archaeal community structure and functional diversity along a geochemically variable soil profile. *Appl. Environ. Microbiol.* **74**, 1620-1633 (2008).

4 Kim, B.-S., Oh, H.-M., Kang, H. & Chun, J. Archaeal diversity in tidal flat sediment as revealed by 16S rDNA analysis. *J. Microbiol.* **43**, 144-151 (2005).

5 Jiang, H. *et al.* Response of archaeal community structure to environmental changes in lakes on the Tibetan Plateau, northwestern China. *Geomicrobiol. J.* **26**, 289-297 (2009).

6 Jiang, H. *et al.* Microbial diversity in water and sediment of Lake Chaka, an athalassohaline lake in northwestern China. *Appl. Environ. Microbiol.* **72**, 3832-3845 (2006).

7 Yang, H. *et al.* Soil pH impact on microbial tetraether lipids and terrestrial input index (BIT) in China. *Sci. China. Earth. Sci.* **55**, 236-245 (2012).

8 Huang, Q. *et al.* Archaeal and bacterial diversity in hot springs on the Tibetan Plateau, China. *Extremophiles* **15**, 549-563 (2011).

9 Meng, X., Mao, Z., Chen, G., Yang, Y. & Xie, B. Diversity of soil archaea in Tibetan Mila Mountains. *Acta Microbiologica Sinica* **49**, 994-1002 (2009).

10 Kato, S., Itoh, T. & Yamagishi, A. Archaeal diversity in a terrestrial acidic spring field revealed by a novel PCR primer targeting archaeal 16S rRNA genes. *FEMS Microbiol. Lett.* **319**, 34-43 (2011).

11 Singh, S. K., Verma, P., Ramaiah, N., Chandrashekar, A. A. & Shouche, Y. S. Phylogenetic diversity of archaeal 16S rRNA and ammonia monooxygenase genes from tropical estuarine sediments on the central west coast of India. *Res. Microbiol.* **161**, 177-186 (2010).

12 Ren, J., Yan, B. & Hong, K. Comparison of bacterial and archaeal community of mangrove soil under different vegetation in Dongzhaigang, Hainan Island. *Acta Microbiologica Sinica* **52**, 736-743 (2012).

13 Wei, S. *et al.* Diversity and distribution of archaea community along a stratigraphic permafrost profile from Qinghai-Tibetan Plateau, China. *Archaea* **2014,** 240817 (2014).

14 Jiang, L., Zheng, Y., Chen, J., Xiao, X. & Wang, F. Stratification of Archaeal communities in shallow sediments of the Pearl River Estuary, Southern China. *Antonie Van Leeuwenhoek* **99**, 739-751 (2011).

15 Gong, L. *et al.* Methane oxidizing bacterial communities in soil with CH4 seeping in Liangfengya, Chongqing. *Quaternary Sciences* **33**, 58-67 (2013).

16 LE, B. P., Gerard, M., Christa, S. & Maria, T. Seasonal and vertical distribution of putative ammonia-oxidizing thaumarchaeotal communities in an oligotrophic lake. *FEMS Microbiol. Ecol.* **83**, 515-526 (2013).
